# Prognostic significance of maximum primary tumor diameter in nasopharyngeal carcinoma

**DOI:** 10.1186/1471-2407-13-260

**Published:** 2013-05-27

**Authors:** Shao-Bo Liang, Yan-Ming Deng, Ning Zhang, Rui-Liang Lu, Hai Zhao, Hai-Yang Chen, Shao-En Li, Dong-Sheng Liu, Yong Chen

**Affiliations:** 1 Radiotherapy Department of Nasopharyngeal Carcinoma, Cancer Center, The First People’s Hospital of Foshan, 81 Lingnan Street North, Foshan, People’s Republic of China; 2 State Key Laboratory of Oncology in South China, Department of Radiation Oncology, Sun Yat-sen University Cancer Center, 651 Dongfeng Road East, Guangzhou, People’s Republic of China; 3 Chemotherapy Department of Head & Neck & Chest Carcinoma, Cancer Center, The First People’s Hospital of Foshan, 81 Lingnan Street North, Foshan, People’s Republic of China; 4 Department of Imaging Diagnosis, The First People’s Hospital of Foshan, 81 Lingnan Street North, Foshan, People’s Republic of China; 5 Department of Medical Statistics, The First People’s Hospital of Foshan, 81 Lingnan Street North, Foshan, People’s Republic of China

**Keywords:** Nasopharyngeal carcinoma, Magnetic resonance imaging, Maximum primary tumor diameter, TNM stage, Survival

## Abstract

**Background:**

To evaluate the prognostic value of maximum primary tumor diameter (MPTD) in nasopharyngeal carcinoma (NPC).

**Methods:**

Three hundred and thirty-three consecutive, newly-diagnosed NPC patients were retrospectively reviewed. Kaplan-Meier analysis and the log-rank test were used to estimate overall survival (OS), failure-free survival (FFS), distant metastasis-free survival (DMFS) and local relapse-free survival (LRFS). Cox proportional hazards regression analysis was used to assess the prognostic value of MPTD.

**Results:**

Median follow-up was 66 months (range, 2–82 months). Median MPTD in stage T1, T2, T3 and T4 was 27.9, 37.5, 45.0 and 61.3 mm, respectively. The proportion of T1 patients with a MPTD ≤ 30 mm was 62.3%; 72% and 62.9% of T2 and T3 patients had a MPTD > 30–50 mm, and 83.5% of T4 patients had a MPTD > 50 mm. For patients with a MPTD ≤ 30 mm, > 30–50 mm and > 50 mm, the 5-year OS, FFS, DMFS and LRFS rates were 85.2%, 74.2% and 56.3% (*P* < 0.001); 87%, 80.7% and 62.8% (*P* < 0.001); 88.7%, 86.4% and 72.5% (*P* = 0.003); and 98.2%, 93.2% and 86.3% (*P* = 0.012), respectively. In multivariate analysis, MPTD was a prognostic factor for OS, FFS and DMFS, and the only independent prognostic factor for LRFS. For T3-T4 patients with a MPTD ≤ 50 mm and > 50 mm, the 5-year OS, FFS and DMFS rates were 70.4% vs. 58.4% (*P* = 0.010), 77.5% vs. 65.2% (*P* = 0.013) and 83.6% vs. 73.6% (*P* = 0.047), respectively. In patients with a MPTD ≤ 30 mm, 5-year LRFS in T1, T2, T3 and T4 was 100%, 100%, 88.9% and 100% (*P* = 0.172).

**Conclusions:**

Our data suggest that MPTD is an independent prognostic factor in NPC, and incorporation of MPTD might lead to a further refinement of T staging.

## Background

The choice of treatment strategies for cancer patients is based on accurate judgment of the severity of disease and prognosis. The current 7th edition of the American Joint Committee on Cancer (AJCC) staging system for nasopharyngeal carcinoma (NPC) is now being used widely throughout the world. In this system, the criteria for assessment of T stage are based on the invasion of anatomical sites and cranial nerve paralysis, but do not include an assessment of tumor load [1]. Tumor load is an important factor which influences the prognosis of patients with NPC [2]. The indicators which can reflect tumor load in NPC include tumor volume, clonogen number, plasma levels of viral Epstein-Barr deoxyribonucleic acid (DNA), and so on [2-4].

Tumor volume is commonly used to represent the tumor load. Previous research demonstrated that the primary tumor volume (PTV) could serve as an important prognostic factor in NPC [5,6]. Chua et al. reported that the PTV represented an independent prognostic factor for local control, which appeared to be more predictive than Ho’s T stage classification [7]. Sze et al. also discovered that the PTV was a highly significant factor for predicting local control in NPC. The risk of local failure was estimated to increase by 1% for every 1 cm^3^ increase in the PTV [8]. These studies were both based on CT techniques. However, MRI offers improved soft tissue contrast resolution, compared to CT. Indeed, recent data suggests that MRI is the imaging modality of choice for the clinical investigation of local disease in NPC patients [9,10]. In fact, Chong et al. also measured PTV using MRI, and their results indicated that it might be possible to incorporate tumor volume as an additional prognostic factor within the existing TNM system [11].

Measurement of PTV by conventional manual tracing is so complicated and time consuming that it is generally not suitable for clinical practice, and also violates the simple principles which staging systems should follow. This raises the question of whether another simple method related to the tumor load exists to predict the prognosis of NPC instead of tumor volume. Maximum primary tumor diameter (MPTD) is widely used in the TMN staging of head-and-neck cancers, such as oral carcinoma, oropharyngeal carcinoma and hypopharyngeal carcinoma [1]. Furthermore, the response evaluation criteria in solid tumors (RECIST) use minification of the maximum tumor diameter to reflect treatment effect [12]. Although tumor volume provides a more accurate assessment of the size of a tumor than the maximum tumor diameter, the maximum tumor diameter can be rapidly and simply measured, suggesting that maximum tumor diameter may be more suitable for the TNM staging system in routine clinical practice.

There are no relevant reports on the prognostic value of the MPTD in NPC. Therefore, we initiated a retrospective study of a large cohort of patients to evaluate the prognostic value of the MPTD in NPC.

## Methods

### Study population

This retrospective study was approved by the ethics committee of the First People’s Hospital of Foshan, Foshan, China. All patients with NPC treated by definitive-intent radiation therapy at the First People’s Hospital of Foshan between October 2005 and August 2007 were eligible, and 333 patients with newly diagnosed, nonmetastatic, histologically-proven NPC were enrolled in the study. There were 239 males and 94 females (male: female ratio, 2.5:1). The median age was 48 years (range, 16–90 years). Histologically, 98.2% of the patients had non-keratinizing NPC, 0.3% had keratinizing NPC, and the remainder (1.5%) had other types. All patients underwent a pretreatment evaluation that included a complete history, physical and neurological examinations, hematology and biochemistry profiles, MRI scan of the nasopharynx and neck, chest radiography and abdominal sonography. Medical and imaging records were retrospectively reviewed, and all patients were restaged according to the 7th edition of the AJCC. The TNM stage distribution of all patients was 15.9% for T1, 15.0% for T2, 45.3% for T3, and 23.7% for T4; 6.9% for N0, 38.4% for N1, 48.3% for N2, and 6.3% for N3; 2.7% for stage I, 12.9% for stage II, 55.9% stage III, and 28.5% stage IVA-B.

### Imaging protocol

All patients underwent MRI using a 1.0 Tesla system (Siemens Magnetom Impact, Germany). The area from the suprasellar cistern to the inferior margin of the sternal end of the clavicle was examined using a head and neck segregate coil. T1-weighted fast spin-echo images in the axial, coronal and sagittal planes (repetition time of 500 ms and echo time of 12 ms), and T2-weighted fast spin-echo MR images in the axial plane (repetition time of 3304 ms and echo time of 96 ms) were obtained before the injection of contrast material. After intravenous injection of gadopentetate dimeglumine (Gd-DTPA; 0.1 mmol/kg body weight; Magnevist, Schering, Berlin, Germany), spin-echo T1-weighted axial and sagittal sequences, and spin-echo T1-weighted fat-suppressed coronal sequences were performed sequentially, using similar parameters to those used before Gd-DTPA injection. We used a section thickness of 5 mm, and a 256 × 256 matrix size.

### Image assessment

Two radiologists with a clinical focus on head and neck cancer and certifications for professional diagnostic imaging in China, who have been on staff for 10 years, evaluated the MR images separately. Any disagreements were resolved by consensus every two weeks. Tumors and soft tissue had intermediate signal intensity on pre-Gd-DTPA-T1 and T2-weighted images and enhanced intensity on post-Gd-DTPA T1-weighted images, replacing the normal anatomy of the structure. The method applied to measure MPTD was as follows: firstly, MPTD was measured on post-Gd-DTPA T1-weighted images. Secondly, tumor signal was not interrupted, but continuous on the maximum diameter. Finally, the maximum diameters in the axial, coronal and sagittal planes were measured separately; the largest value was recorded as the MPTD.

### Treatment

All patients were treated by definitive-intent radiation therapy. Two hundred and four patients (61.3%) were treated with conventional 2-dimensional radiotherapy (2-DRT), and 129 patients (38.7%) with 3-dimensional conformal radiotherapy (3-DCRT). The accumulated doses were 68–70 Gy to the gross tumor, 60–62 Gy to the involved areas of the neck and 50 Gy to the uninvolved areas. Additional boosts to the parapharyngeal space, skull-base and primary or nodal sites could be given if indicated, and did not exceed 16 Gy.

Platinum-based induction, concomitant or adjuvant chemotherapy was administered to 184 patients with Stage III or Stage IVa-b disease (classified as T3-T4 or N2-N3). Of these patients, 131 patients received concurrent chemotherapy, 49 patients received induction chemotherapy and 4 patients received adjuvant chemotherapy. The remaining patients with advanced stage disease did not receive chemotherapy due to advanced age, heart disease, hepatitis, severe diabetes, inadequate renal function, patient refusal or economic problems. When possible, salvage treatments (including afterloading, surgery and chemotherapy) were provided in the event of documented relapse or when the disease persisted despite therapy.

### Statistical analysis

Patients were assessed every two months during the first year, every three months for the next two years, and every six months thereafter until death. The median follow-up period for the whole group was 66 months (range, 2–82 months).

All events were measured from the date of commencement of treatment. The following end points (time to the first defining event) were assessed: overall survival (OS), failure-free survival (FFS), local relapse-free survival (LRFS) and distant metastasis-free survival (DMFS). Local recurrence was established by fiberoptic endoscopy and biopsy and/or MRI. Distant metastases were diagnosed based on clinical symptoms, physical examination and imaging methods including chest X-ray, bone scan, CT scan and abdominal sonography.

All statistical analyses were performed using the Statistical Package for the Social Sciences software version 12.0 (SPSS; Chicago, IL, USA). The actuarial rates were calculated by the Kaplan-Meier method, and the differences were compared using the log-rank test. Multivariate analyses with the Cox proportional hazards model were used to test for independent significance by backward elimination of insignificant explanatory variables. Demographic characteristics (age, sex) were introduced into the models as covariates for all statistical tests. The chi-square test was used to analyze the relationship between MPTD and T stage. The criterion for statistical significance was set at α = 0.05; *P* values were based on two-sided tests.

## Results

### Distribution of MPTD by T stage

**Figure 1:**
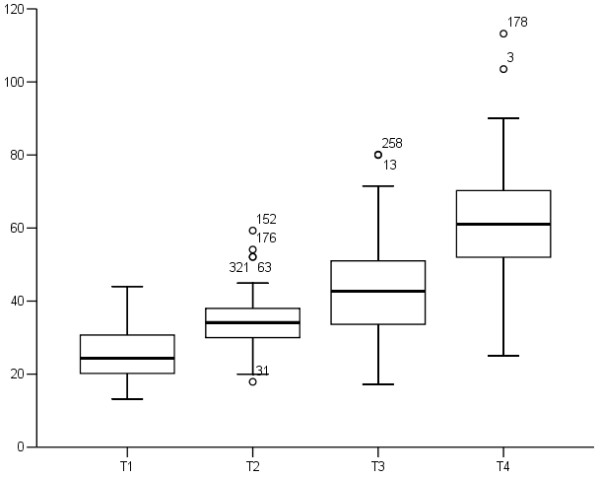
Distribution of maximum primary tumor diameter by T stage in 333 nasopharyngeal carcinoma patients.

The distribution of MPTD by T stage is presented in Figure 1. The median MPTD was 27.9 mm (range, 14.3-45 mm) in Tl, 37.5 mm (range, 22–60.1 mm) in T2, 45.0 mm in T3 (range, 27.1-80.1 mm), and 61.3 mm in T4 (range, 25–113.2 mm). On the basis of patient distribution and survival curves, the patients were divided into three subgroups: MPTD ≤ 30 mm, > 30–50 mm, and > 50 mm. Of the 333 patients, 16.2% had a MPTD ≤ 30 mm; 48.6%, > 30–50 mm; and 35.1%, > 50 mm.

The frequencies of MPTD were superimposed on the different T stages; larger MPTDs were more frequent in patients with higher stage disease (*P* < 0.001, Table 1). In the T1 stage subgroup, 62.3% of the patients had a small MPTD (≤ 30 mm), whereas 72.0% and 62.9% of the T2 and T3 stage patients respectively had a moderate MPTD (> 30–50 mm), and 83.5% of the T4 stage patients had a large MPTD (> 50 mm).

**Table 1:** Distribution of maximum primary tumor diameter by T stage in 333 nasopharyngeal carcinoma patients

**Maximum primary tumor diameter**	**Patients, n (%)**			
	**T1**	**T2**	**T3**	**T4**
≤ 30 mm	33 (62.3%)	10 (20.0%)	9 (6.0%)	2 (2.5%)
> 30–50 mm	20 (37.7%)	36 (72.0%)	95 (62.9%)	11 (13.9%)
> 50 mm	0 (0.0%)	4 (8.0%)	47 (31.1%)	66 (83.5%)

*P* < 0.001, Chi-square test.

### Association of MPTD with prognosis in NPC

**Figure 2:**
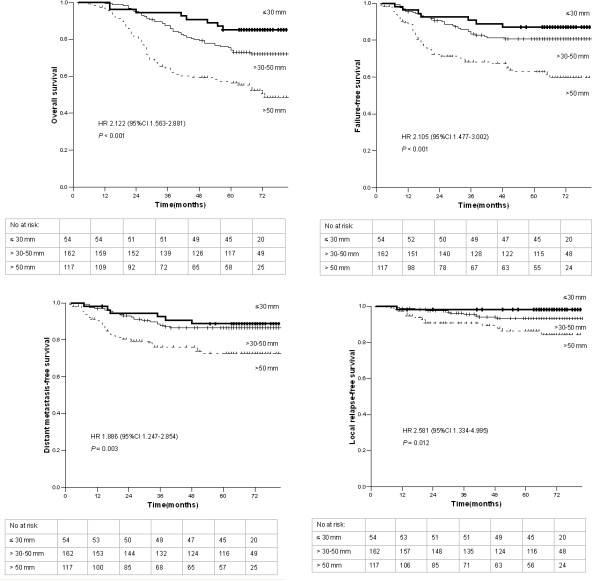
**Overall survival, failure-free survival, distant metastasis-free survival and local relapse-free survival of 333 patients with nasopharyngeal carcinoma stratified by maximum primary tumor diameter.** Hazard ratios (HRs) were calculated with the unadjusted Cox proportional hazards model; p values were calculated with the unadjusted log-rank test.

Univariate analysis of the association of MPTD with prognosis was performed, and the results are shown in Figure 2. The 5-year OS rates for patients with a MPTD ≤ 30 mm, > 30–50 mm and > 50 mm were 85.2%, 74.2% and 56.3%, respectively. The differences among these rates were highly significant (HR = 2.122, 95% CI = 1.563-2.881; *P* < 0.001; Figure 2). The FFS rates for patients with a MPTD ≤ 30 mm, > 30–50 mm and > 50 mm were 87.0%, 80.7% and 62.8% (HR = 2.105, 95% CI = 1.477-3.002; *P* < 0.001; Figure 2). Similarly, the DMFS and LRFS rates for patients with a MPTD ≤ 30 mm, > 30–50 mm and > 50 mm were 88.7%, 86.4% and 72.5% (HR = 1.886, 95% CI = 1.247-2.854; *P* = 0.003; Figure 2), and 98.2%, 93.2% and 86.3% (HR = 2.581, 95% CI = 1.334-4.995; *P* = 0.012; Figure 2), respectively.

To adjust for prognostic factors, the following parameters were introduced into the Cox regression model: age (≤ 45 vs. > 45 years), sex, chemotherapy (yes vs. no) and radiation technique (2-DRT vs. 3-DCRT). For analysis of OS, FFS and DMFS, the following additional covariates were introduced into the model: T stage (T1 vs. T2 vs. T3 vs. T4), N stage (N0 vs. N1 vs. N2 vs. N3), and MPTD (≤ 30 mm vs. > 30–50 mm vs. > 50 mm). For analysis of LRFS, the following additional covariates were taken into account: intracranial extension, skull base erosion, nasal extension, oropharyngeal extension, paranasopharyngeal extension and MPTD. The results from the final models for OS, FFS, DMFS and LRFS are summarized in Table 2. In the model for OS, age, N stage and MPTD were unfavorable prognostic factors. The FFS and DMFS models showed that N stage and MPTD were independent prognostic factors. With regard to LRFS, MPTD was the only independent prognostic factor.

**Table 2:** Summary of multivariate analyses of prognostic factors in 333 nasopharyngeal carcinoma patients

**Endpoint**	**Variable**	**Estimate**	**HR**†	95% CI*	***P *value**^**‡**^
OS	Age	-0.403	0.668	0.450-0.993	0.046
	N stage	0.312	1.366	1.037-1.799	0.027
	MPTD	0.720	2.054	1.509-2.794	< 0.001
FFS	N stage	0.527	1.694	1.218-2.357	0.002
	MPTD	0.713	2.040	1.426-2.919	< 0.001
DMFS	N stage	0.628	1.873	1.269-2.766	0.002
	MPTD	0.563	1.757	1.155-2.671	0.008
LRFS	MPTD	0.955	2.598	1.342-5.033	0.005

†HR: Hazard ratio from Cox proportional hazards model; *CI, confidence interval; ^**‡**^*P* values were calculated using an adjusted Cox proportional-hazards model. OS, overall survival; FFS, failure-free survival; DMFS, distant metastasis-free survival; LRFS, local relapse-free survival; MPTD, maximum primary tumor diameter. The following parameters were included in the model as the covariates for each analysis: age (≤ 45 vs. > 45 years), sex, chemotherapy (yes vs. no) and radiation technique (2-DRT vs. 3-DCRT). For analysis of OS, FFS and DMFS, the following additional covariates were introduced into the model: T stage (T1 vs. T2 vs. T3 vs. T4), N stage (N0 vs. N1 vs. N2 vs. N3), and MPTD (≤ 30 mm vs. > 30–50 mm vs. > 50 mm). For analysis of LRFS, the following additional covariates were taken into account: intracranial extension, skull base erosion, nasal extension, oropharyngeal extension, paranasopharyngeal extension and MPTD. We have only presented the results for MPTD and other statistically significant variables.

### The prognostic significance of MPTD in stage T3-4 patients

**Figure 3:**
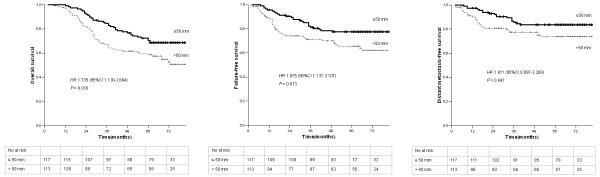
**Overall survival, failure-free survival and distant metastasis-free survival of 230 patients with T3-T4 stage nasopharyngeal carcinoma stratified by maximum primary tumor diameter.** Hazard ratios (HRs) were calculated with the unadjusted Cox proportional hazards model; p values were calculated with the unadjusted log-rank test.

Among the 230 patients with T3-T4 stage disease, 4.8% patients had a MPTD ≤ 30 mm; 46.1%, > 30–50 mm; and 35.1%, > 50 mm. Due to the small number of patients with a MPTD ≤ 30 mm, we divided the patients into two groups (MPTD ≤ 50 mm and MPTD > 50 mm). The 5-year OS rates of 70.4% for the group with a MPTD ≤ 50 mm and 58.4% for the group with a MPTD > 50 mm were significantly different (HR = 1.735, 95% CI = 1.130-2.664; *P* = 0.010; Table 3; Figure 3). The 5-year FFS and DMFS rates also differed significantly (77.5% vs. 65.2%, *P* = 0.013; Table 3; Figure 3; and 83.6% vs. 73.6%, *P* = 0.047; Table 3; Figure 3; respectively). However, the 5-year LRFS rates were not significantly different (91.5% vs. 87.9%, *P* = 0.261; Table 3).

**Table 3:** Summary of survival outcomes in T3-T4 nasopharyngeal carcinoma patients with a maximum primary tumor diameter (MPTD) ≤ 50 mm and MPTD > 50 mm

**Variable**	**MPTD ≤ 50 mm**	**MPTD > 50 mm**	**Hazard ratio**^**†**^	***P *value**^**‡**^
	**(*N*** **= 117)**	**(*N*** **= 113)**	(95% CI*)	
5-yr OS rate	70.4%	58.4%	1.735 (1.130-2.664)	0.010
5-yr FFS rate	77.5%	65.2%	1.875 (1.131-3.107)	0.013
5-yr DMFS rate	83.6%	73.6%	1.811 (0.997-3.289)	0.047
5-yr LRFS rate	91.5%	87.9%	1.634 (0.688-3.882)	0.261

†Hazard ratios were calculated using the unadjusted Cox proportional-hazards model; *CI, confidence interval; ‡P values were calculated by the unadjusted log-rank test. OS, overall survival; FFS, failure-free survival; DMFS, distant metastasis-free survival; LRFS, local relapse-free survival; MPTD, maximum primary tumor diameter.

### Local control in patients with a small MPTD

The prognosis of patients with a small MPTD (≤ 30 mm) was analyzed according to T stage. A total of 54 patients were categorized with a small MPTD: 33 (61.1%) stage T1, 10 (18.5%) stage T2, 9 (16.7%) stage T3, 2 (3.7%) stage T4 patients. Among these patients with a small MPTD, the 5-year LRFS rates for stages T1, T2, T3 and T4 were 100%, 100%, 88.9% and 100%, respectively (*P* = 0.172). Thus, when early stage and advanced stage NPC patients were compared, a small MPTD led to excellent local control, regardless of T stage.

## Discussion

The 5-year overall survival rate for NPC has increased from approximately 50% to 75% over the last ten years [13-15]. Advances in diagnostic technology, radiotherapy techniques and the introduction of combined chemotherapy are obvious, important contributors to this achievement [16-19]. The staging system was originally designed to help predict prognosis, define treatment strategies, and evaluate the outcome of treatment. Significant efforts have been made to improve the staging system for NPC, as it is well recognized that the current system has some limitations.

### Image assessment

CT was widely used for diagnostic imaging in NPC before the application of MRI; however, MRI enables enhanced soft tissue contrast resolution and multiplanar imaging capability. MRI offers several advantages over CT for the assessment of local disease in NPC patients, including more accurate definition of early invasion outside the nasopharynx, improved differentiation of the retropharyngeal nodes from the primary tumor, as well as more accurate assessment of the parapharyngeal space, skull base, paranasal sinus and cranial invasion [9]. In light of these developments, previous studies of the prognostic value of primary tumor size in NPC, which were based on CT imaging, have a number of limitations. Therefore, this study was designed to evaluate the prognostic value of MPTD, measured from MR imaging, in NPC patients.

The retropharyngeal lymph node chain is located close to the nasopharynx, which is the first step of node metastasis in NPC. Tang et al. reported that the incidence of retropharyngeal lymph node metastasis was 73.5% in 924 consecutive patients with newly diagnosed, untreated, non-metastatic NPC, as diagnosed by MRI [20]. Chua et al. included the retropharyngeal lymph nodes that were embedded in the primary tumor in their measurement of tumor volume [7]. In current study, we excluded the retropharyngeal nodes from the volume of the primary tumor during measurement of the maximum primary tumor diameter for the following reasons. Firstly, MRI, which can provide a clear distinction between the retropharyngeal nodes and primary tumor, has been widely used in the staging of NPC [21]. Secondly, retropharyngeal node involvement has already been reported to significantly affect DMFS in NPC, leading to the reclassification of retropharyngeal node involvement as N1, instead of T2, disease [1,20].

### Comparison of MPTD by T stage

In 1999, Willner et al. reported that tumor volume was an important factor which influenced local control in NPC. Their results suggested that the clinically observed smooth dose–response relationships in NPC might be explained by interindividual tumor volume heterogeneity [22]. Subsequently, a number of studies confirmed that PTV was a more important prognostic factor in NPC than T stage [7,8,23]. However, there are no reports on the prognostic value of the MPTD in NPC. In our study, large MPTDs were frequently observed in advanced T stage disease. However, MPTD varied largely within each T stage, and the range of MPTDs overlapped between different T stages. Similar results were observed in studies of the correlation between PTV and T stage, as PTV also varied in each T stage and overlapped between different T stages [7,8]. This observation indicates that there might be some limitations of the current NPC staging system to separate patients with a large and small tumor bulk. Furthermore, MPTD was the only independent predictor of LRFS. In agreement with this observation, Chang et al. reported that PTV represented a more important prognostic factor for treatment outcome in advanced-T staged NPC [24]. Tumor volume and diameter are both indexes which could be used to represent the tumor bulk, and might be superior to T stage for the prediction of local control in NPC patients. Notably, the local control rate in patients with a small MPTD was excellent, and was unaffected by T stage. Chua et al. also reported that there were no significant differences in the local control rates of patients with a small PTV (< 20 cm^3^) according to T stage [7]. Therefore, if a measure of tumor bulk, including either the MPTD or PTV is incorporated into T staging, then the TNM staging system could better predict local control in NPC.

### Other prognostic value of MPTD

In this study, the 5-year OS, FFS and DMFS rates were significantly different in patient subgroups with different MPTDs (≤ 30 mm vs. > 30–50 mm vs. > 50 mm; all *P* < 0.05). Additionally, MPTD was an independent predictor of OS, FFS and DMFS in multivariate analysis. Furthermore, for stage T3-T4 patients, the group with a MPTD ≤ 50 mm had significantly better OS, FFS and DMFS rates (all *P* < 0.05) while comparing with another group with a MPTD > 50 mm. Therefore, the patients’ prognoses became poorer as MPTD increased. In a recent study by Guo et al., a larger PTV (≥ 19 ml vs. < 19 ml) also had an unfavorable impact on OS, FFS, DMFS and LRFS in patients with NPC treated by intensity modulated radiotherapy (IMRT) [25].

Taken together, previous research and this study clearly demonstrate that MPTD and PTV are both good prognostic indicators in NPC [7,8]. However, compared to PTV, MPTD has several advantages. Firstly, MPTD is easier and quicker to measure; therefore would be convenient for TNM staging in routine clinical practice. Secondly, accurate measurement of the PTV requires a calculation of tumor volume from a three-dimensional perspective, but MPTD does not. Thirdly, the RECIST criteria already use minification of maximum tumor diameter to evaluate the effects of treatment. Therefore, the addition of MPTD to the TNM staging system may improve prognostic ability, treatment selection and the evaluation of treatment in NPC patients.

### Limitations of this study

Firstly, treatment variability might be one of the limitations in this study. Due to limited medical resources, 2-DRT and 3D-CRT were used instead of IMRT. Although excellent local control can be achieved in NPC by IMRT, distant metastasis remains the major cause of disease failure [14,16,26]. Furthermore, most patients with advanced disease in this study received chemotherapy, though some patients with advanced disease did not receive chemotherapy (due to advanced age, heart disease, hepatitis, severe diabetes, inadequate renal function, patient refusal or economic problems). However, when included as covariates, neither the radiotherapy technique nor chemotherapy was independent prognostic factors in the multivariate analyses. Secondly, our study was a retrospective study, and the conclusions need to be confirmed by future prospective studies.

## Conclusions

This study is the first attempt to evaluate the prognostic value of MPTD in NPC. Our analyses demonstrate that MPTD is an independent prognostic factor for OS, FFS, DMFS and LRFS in patients with NPC. Addition of MPTD might help to refine the prognostic ability of the current staging system for NPC and assist with selection of treatment strategies.

## Competing interests

The authors indicate no actual or potential conflicts of interest exist.

## Authors’ contributions

S-BL and Y-MD participated in literature research, study design, data collection, data analysis, interpretation of findings and the draft of the manuscript. NZ, H-YC and S-EL carried out the data collection. R-LL and HZ reviewed MR images. D-SL performed the statistical analysis. YC contributed with study design, data collection, interpretation of findings and critical edit of the manuscript. All authors read and approved the final manuscript.

## Pre-publication history

The pre-publication history for this paper can be accessed here:

http://www.biomedcentral.com/1471-2407/13/260/prepub
